# A Distinct T Follicular Helper Cell Subset Infiltrates the Brain in Murine Neuropsychiatric Lupus

**DOI:** 10.3389/fimmu.2018.00487

**Published:** 2018-03-13

**Authors:** Shweta Jain, Ariel Stock, Fernando Macian, Chaim Putterman

**Affiliations:** ^1^Division of Rheumatology, Albert Einstein College of Medicine, Bronx, NY, United States; ^2^Department of Microbiology and Immunology, Albert Einstein College of Medicine, Bronx, NY, United States; ^3^Department of Pathology, Albert Einstein College of Medicine, Bronx, NY, United States

**Keywords:** systemic lupus erythematosus, neuropsychiatric lupus, choroid plexus, T follicular helper cells, MRL/lpr

## Abstract

Neuropsychiatric symptoms in systemic lupus erythematosus (SLE) are not uncommon, yet the mechanisms underlying disease initiation and progression in the brain are incompletely understood. Although the role of T cells in other lupus target organs such as the kidney is well defined, which T cells contribute to the pathogenesis of neuropsychiatric SLE is not known. The present study was aimed at characterizing the CD4 T cell populations that are present in the choroid plexus (CP) of MRL/MpJ-fas*lpr* mice, the primary site of brain infiltration in this classic lupus mouse model which exhibits a prominent neurobehavioral phenotype. T cells infiltrating the CP of MRL/MpJ-fas*lpr* mice were characterized and subset identification was done by multiparameter flow cytometry. We found that the infiltrating CD4 T cells are activated and have an effector phenotype. Importantly, CD4 T cells have a T follicular helper cell (T_FH_) like phenotype, as evidenced by their surface markers and signature cytokine, IL-21. In addition, CD4 T_FH_ cells also secrete significant levels of IFN-γ and express Bcl-6, thereby conforming to a potentially pathogenic T helper population that can drive the disease progression. Interestingly, the regulatory axis comprising CD4 T regulatory cells is diminished. These results suggest that accumulation of CD4 T_FH_ in the brain of MRL/MpJ-fas*lpr* mice may contribute to the neuropsychiatric manifestations of SLE, and point to this T cell subset as a possible novel therapeutic candidate.

## Introduction

Signs and symptoms of primary neuropsychiatric disease in systemic lupus erythematosus (SLE) (i.e., due to lupus itself rather than iatrogenic or other causes) include a broad range of clinical manifestations, such as cognitive disorders, seizures, stroke, psychosis, and depression (1). The immunopathogenesis of neuropsychiatric systemic lupus erythematosus (NPSLE) is complex and multifactorial, involving adhesion molecule upregulation together with inflammatory cytokines and/or neuropathic autoantibodies that reach the brain through abnormally permeable brain barriers, leading to neuronal damage (2, 3). The presence of affinity matured autoantibodies along with lymphocyte infiltrates in brain autopsy tissue from SLE patients with neurological disease support a role for T cells in the pathogenesis of NPSLE (4).

The role of T cells in the immunopathogenesis of SLE outside the central nervous system (CNS) has been widely studied. T cells from SLE patients display aberrant T cell signaling, activation, and function (5). In addition, infiltrating T cells, including oligoclonal T cells, are found in several tissues such as salivary glands and kidneys (6). Moreover, T cell transcriptomic data from lupus nephritis patients indicate widespread induction of inflammatory genes and metabolic pathways such as glycolysis and increased oxidative phosphorylation (7, 8). In addition to abnormal T cell function, SLE is also characterized by differential expression of various T cell subsets. Studies in lupus report an increased accumulation of effector/memory CD4^+^ T cells, Th17 cells, T follicular helper cells (T_FH_), γδ T cells, and double negative (DN) T cells in the blood, lymphoid tissue, and target organs (6, 9–11). On the other hand, suppressive T cell subsets such as T regulatory cells (Tregs) are decreased in SLE, contributing to an imbalance in immune homeostasis (12).

The MRL/MpJ-fas*^lpr^* (MRL/lpr) mouse strain is a widely studied spontaneous lupus model with many parallels with human SLE (13). In particular, female MRL/lpr mice exhibit neurobehavioral changes that resemble human NPSLE, including depression-like behavior and cognitive deficits which are evident by 16 weeks of age (14). In addition, MRL/lpr mice have aberrant IL-2 function and display severe T cell driven lymphadenopathy that is largely attributable to expansion of DN T cells (15, 16). However, although T cells can be found scattered throughout the brain of MRL/lpr mice, they are particularly concentrated in an area of one of the barriers between the brain and the systemic circulation, i.e., the choroid plexus (CP) or blood cerebrospinal fluid barrier. Moreover, experimental manipulations which decrease T cell accumulation in the CP attenuate the neurobehavioral phenotype (17). However, there are no published reports describing careful identification and subset characterization of brain infiltrating CD4^+^ T cells in murine lupus.

We report here that CD4^+^ T cells infiltrating the CP of MRL/lpr mice are activated and have a functional effector phenotype. We also demonstrate that CD4^+^ T cells secrete high levels of IFN-γ and IL-21, and express signature T_FH_ markers including ICOS, PD1, CXCR5, and Bcl6. Moreover, regulatory cells such as Tregs and T follicular regulatory cells (Tfr) were only rarely found among the CP infiltrating T cells. These data strongly support a role for pathogenic CD4^+^ T subsets in the pathogenesis of neuropsychiatric lupus, and encourage the development of targeted therapies to address lupus involving the CNS.

## Materials and Methods

### Mice

The 8–10–week-old MRL/lpr (stock # 000485) and MRL/+ (stock # 000486) mice were purchased from The Jackson Laboratories (Bar Harbor, ME, USA). Female mice were used unless otherwise specified. NPSLE manifestations are absent in the congenic MRL/+ strain and more prominent in female than in male MRL/lpr mice (18, 19), and CP infiltrating T cells were found to be rare or diminished in the non-autoimmune control MRL/+ strain and in age matched male MRL/lpr mice, respectively (see below). Hence, MRL/+ or male MRL/lpr mice were used as controls in some experiments. Mice were housed in the animal facility of Albert Einstein College of Medicine until they were 16–18 weeks of age, at which time the MRL/lpr strain exhibits a profound neurobehavioral phenotype including cognitive deficits and depressive like behavior (20–22). All animal studies were performed under protocols approved by the Institutional Animal Care and Use Committee of the Albert Einstein College of Medicine.

### Tissue Isolation

Spleens and brains were harvested from mice after transcardial perfusion with ice cold HBSS (Cellgro, Manassas, VA, USA). Single cell suspensions of spleens were prepared by mechanical disruption, and residual red blood cells were lysed using ACK lysis buffer (Quality Biologicals, Gaithersburg, MD, USA) for 5 min at room temperature. The CP was isolated from the brain by careful dissection and the tissue was dissociated in 0.25% trypsin–2.21 mM EDTA (Cellgro) for 30 min at 37°C. Cells were washed twice with ice cold HBSS supplemented with 2% heat inactivated fetal bovine serum (GIBCO, Auckland, New Zealand) and then used for downstream applications.

Brain tissue devoid of CP [ex-choroid plexus (ex-CP)] was dissociated in a digestion buffer containing Liberase TL (3.25 U/ml; Sigma, St. Louis, MO, USA), DNase I (0.1 mg/ml; Sigma), and BSA (1%; Sigma) in HBSS (with Ca^2+^ and Mg^2+^; GIBCO) for 30 min at 37°C. EDTA (1 mM; Sigma) was added to the solution and the cell suspension was filtered through a 40 μm filter (BD, San Diego, CA, USA) and centrifuged at 1,500 rpm for 15 min at 4°C. Isotonic Percoll (30%) (GE Healthcare, Uppsala, Sweden) was added to the pellet, and the suspension carefully layered onto 70% of isotonic Percoll. The gradient was centrifuged for 30 min at 20°C and the cells at the 70–30% interphase were collected, washed, and used for downstream applications.

### Immunofluorescent Staining

Formalin fixed paraffin embedded sections were deparaffinized in xylene and rehydrated in graded ethanol concentrations. Sections were blocked in 20% normal horse serum in PBS and incubated in 1:100 rat antimouse CD4 in 2% normal horse serum in PBS (eBioscience) overnight at room temperature. Sections were washed in PBS followed by staining with secondary donkey antirat for 1 h at room temperature. After washing, sections were counter stained with DAPI and mounted with fluoromount-G. Slides were then visualized with a Thermo Scientific EvosFL Auto 2.

### Flow Cytometry

For surface staining, Fc receptors (FcRs) were blocked using an antimouse CD16/32 antibody (BD) for 15 min on ice, and standard multiparameter flow cytometric analyzes was performed by staining cells with the antibodies listed in Table 1. Data were collected using FACS Diva software on a BD LSR II analyzer (BD), and analyzed using FlowJo_V10 software (TreeStar, Ashland, OR, USA). Doublet discrimination was performed and viable cells were analyzed using either 7AAD (Invitrogen, Carlsbad, CA, USA) or the fixable viability dye (Biolegend, San Diego, CA, USA) exclusion method. For intracellular cytokine staining, cells were initially stimulated with a cell activation cocktail (Biolegend) (6 h, 37°C, 5% CO_2_) and staining for IFN-γ, IL-21, IL-17, and IL-4 was performed using the BD CytoFix/Perm Kit following the manufacturer’s instructions. Staining for intracellular IL-21 was done using an IL-21R/Fc chimera (R&D Systems) and PE-conjugated F(ab′)2 fragment of goat anti–human Fcγ antibody (anti-Fc PE; Jackson ImmunoResearch, West Grove, PA, USA). For all intracellular staining, unstained cells (permeabilized as well as non-permeabilized) as well as fluorescence-minus-one were used to set gates and as negative controls. Staining for transcription factors including FoxP3, Tbet, and Bcl6 was done using the FoxP3 staining kit from eBiosciences (ThermoFisher, Waltham, MA, USA).

**Table 1:** List of antibodies used in flow cytometry.

Antibody	Clone	Fluorophore/format	Company
CD45	30-F11	FITC, PE, Alexa700, APCCy7	BD Biosciences, CA, USA
CD3	17A2	PECy7, PerCP-Cy5.5	BD Biosciences, CA, USA
CD4	RM4-5	APC, FITC, Pacific Blue, PECy7	BD Biosciences, CA, USABiolegend, CA, USA
CD8	53–6.7	PECy7, APCCy7	BD Biosciences, CA, USA
ICOS	7E-17Gg	PE, Biotin	BD Biosciences, CA, USA
PD1	29F.1A12	FITC, APC	BD Biosciences, CA, USABiolegend, CA, USA
CXCR5	L138D7	PerCP-Cy5.5	Biolegend, CA, USA
CD25	PC61	APCCy7, FITC	BD Biosciences, CA, USA
CD62L	MEL-14	APCCy7	BD Biosciences, CA, USA
CD69	H1.2F3	PE	BD Biosciences, CA, USA
CD44	1M7	Pacific Blue	BD Biosciences, CA, USA
IL17A	TC11-18H10.1	FITC	BD Biosciences, CA, USA
IL4	11B11	PE	BD Biosciences, CA, USA
IFNγ	XMG1.2	APC, PerCP-Cy5.5	BD Biosciences, CA, USA
Bcl6	Ig191E/A8	PE	Biolegend, CA, USA
FoxP3	MF-14	Pacific Blue	Biolegend, CA, USA
Fc Block or CD16/32	2.4G2	Purified	BD Biosciences, CA, USA

### Cell Proliferation

Analysis of proliferating CD4^+^ T cells was done using Ki-67 staining, with minor modifications. Briefly, cells were surface stained with an anti-mouse CD4 antibody and fixed by adding 70–80% ice-cold ethanol (dropwise with continuous vortexing). Cells were incubated at −20°C overnight, washed, and stained with either PE-labeled Ki-67 antibody or isotype control IgG1κ antibody (BD). Cells were incubated for 30 min in the dark at room temperature, washed, and analyzed by flow cytometry.

### Cell Sorting and *In Vitro* Stimulation

CD4^+^ T cells were sorted from spleens and CP in complete RPMI media on a BD FACS Aria. Sorted cells were counted and washed in complete RPMI media before plating (in duplicates or triplicates) at a density of 5 × 10^4^ − 2 × 10^5^ cells per well in a 96-well plate precoated with anti-CD3 (5 µg/ml; BD). Cells were suspended in complete RPMI-1640 medium (GE Healthcare Life Sciences, Logan, UT, USA) supplemented with FBS (10%), sodium pyruvate (Cellgro), MEM non-essential amino acids (Hyclone), penicillin–streptomycin (Cellgro), and 2-mercaptoethanol (55 µM; GIBCO). Anti-CD28 antibody (2.5 µg/ml; BD) was added to the cultures and incubated for 48 h at 37°C in the presence of 5% CO_2_. Appropriate unstimulated controls were included as well. Supernatants were collected and analyzed for cytokine levels, whereas cells were harvested and stained for the T cell activation markers listed in Table 1.

### Cytokine Quantitation

Measurement of T helper (Th) cytokines was done in the culture supernatants obtained from *in vitro* cultures using the LEGENDplex Mouse Th cytokine panel (Biolegend). Using this multiplex bead based array, soluble analytes were quantified in the supernatants following the manufacturer’s instructions.

### Statistics

Statistical analysis was done using GraphPad Prism 7 Software. Values in the figures are depicted as mean ± SEM of *n* observations, where *n* is detailed in the figure legends. *p*-Values of ≤0.05 were considered significant.

## Results

### MRL/lpr Lupus Mice Display Prominent T Cell Infiltration in the Brain CP

**Figure 1:**
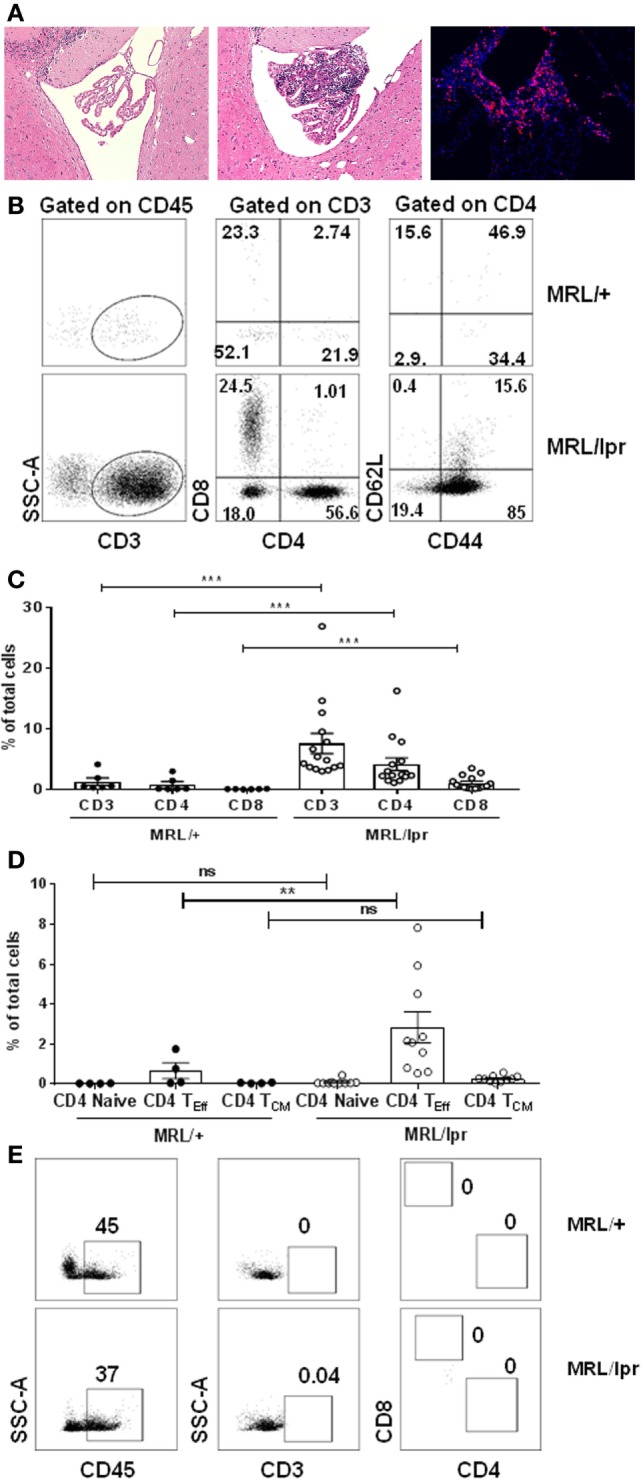
T cells infiltrate the choroid plexus of MRL/lpr mice. **(A)** Representative H&E staining of brain sections from 16-week-old control MRL/+ (left) and MRL/lpr (middle) mice are shown. The right panel demonstrates prominent immunofluorescent staining for CD4 in the choroid plexus of MRL/lpr mice. Magnification: 10×. **(B–E)** Single cell suspensions from choroid plexus of 16–18-week-old female MRL/+ and MRL/lpr were stained for the presence of T cells. **(B)** Comparative representative FACS plots showing CD3, CD4, and CD8 populations. CD4^+^ gated cells were further analyzed for the expression of effector (CD4 T_Eff_; CD4^+^CD44^+^), naive (CD4^+^CD62L^+^), and central memory phenotypes (CD4 T_CM_; CD4^+^CD44^+^CD62L^+^). Values indicate percentage of parent cells. **(C)** Bar graphs depicting mean ± SEM of CD3, CD4, CD8 T cells as percentage of total cells. Each dot represents one mouse. MRL/+ (*n* = 6), and MRL/lpr (*n* = 15). **(D)** Bar graphs depicting CD4 naive, effector, and central memory T cells as percentage of total cells. Each dot represents one mouse. MRL/+ (*n* = 4), and MRL/lpr (*n* = 10). **(E)** Representative FACS plots showing absence of infiltrating T cells in tissue devoid of choroid plexus in MRL/+ and MRL/lpr mice. Data were analyzed using a Mann–Whitney test. **p < 0.01, ***p < 0.001.

Female MRL/lpr mice at ~16 weeks of age exhibit profound neurobehavioral deficits, including depression-like behavior and cognitive (memory) abnormalities that model key manifestations of human disease. MRL/+, the congenic strain, do not have significant neurologic deficits (19, 21, 23–25). Therefore, we began these studies using the congenic non-autoimmune Fas-sufficient MRL/+ strain as an appropriate and commonly used control for the lupus prone MRL/lpr mice. To identify the cells infiltrating the brain of lupus mice which are associated with neuropsychiatric disease, CPs from the brains of transcardially perfused 16–18-week-old female MRL/lpr and MRL/+ control mice were isolated and stained for the presence of T lymphocytes. There was extensive lymphocytic infiltration of the CP at this age in the lupus prone MRL/lpr strain, which was absent in age- and sex-matched MRL/+ control mice (Figure 1A). As seen by immunohistochemical staining, many of the infiltrating cells were T lymphocytes (Figure 1A). T cells were rare in other regions of the brain (data not shown). We confirmed these observations by analysis of single cell suspensions. We found that there was a significant population of CD4^+^ and CD8^+^ T cells in the CP of MRL/lpr mice, which was almost absent in MRL/+ controls (Figures 1B,C). As compared to the DN (CD4^−^CD8^−^) phenotype of T cells expanding in lymphoid tissue and responsible for the profound lymphadenopathy and splenomegaly exhibited by MRL/lpr mice (26), T cells infiltrating the CP were preferentially CD4^+^ or CD8^+^ single positive cells. Of these, more than 70% cells were CD4^+^ T cells. Further analysis of brain infiltrating CD4^+^ T cells revealed that they had a functional effector phenotype. In the CP of MRL/lpr mice there was a substantial fraction of effector CD4^+^ T cells (CD44^+^CD62L^−^), followed by a small fraction of central memory CD4^+^ T cells (CD44^+^CD62L^+^) (Figure 1D). Moreover, few naive CD4^+^ T cells (CD44^−^CD62L^+^) were present (Figure 1D). A similar trend was seen with respect to the proportions of CD8^+^ effector, central memory, and naive T cells infiltrating the CP (Figures S1A,B in Supplementary Material). The presence of increased effector CD4^+^ and CD8^+^ T cells indicates abnormal T cell activity and signaling in the CP of MRL/lpr mice with neuropsychiatric manifestations. While CD8^+^ T cells have also been implicated in SLE (27, 28), the subsequent studies are focused on functional and phenotypic characterization of the more abundant CD4^+^ T cells infiltrating the brains of MRL/lpr mice.

Next, to investigate whether brain infiltrating CD4^+^ T cells were localized primarily in the CP or are present as well elsewhere in the brain, we digested brain tissue of MRL/lpr mice following the removal of the CP (i.e., ex-CP) and analyzed this tissue by flow cytometry. Interestingly, we found that brain tissue devoid of CP showed only rare CD3^+^CD4^+^ or CD3^+^CD8^+^ T cells, despite the presence of CD45^+^ cells (Figure 1E). Taken together, these results indicate that T lymphocytes present in the brain of MRL/lpr lupus mice with neuropsychiatric disease are primarily localized to the CP. In addition, the infiltrating T cells are either CD4^+^ or CD8^+^ T cells displaying an effector phenotype.

### CP Infiltrating CD4^+^ T Cells Are Activated

**Figure 2:**
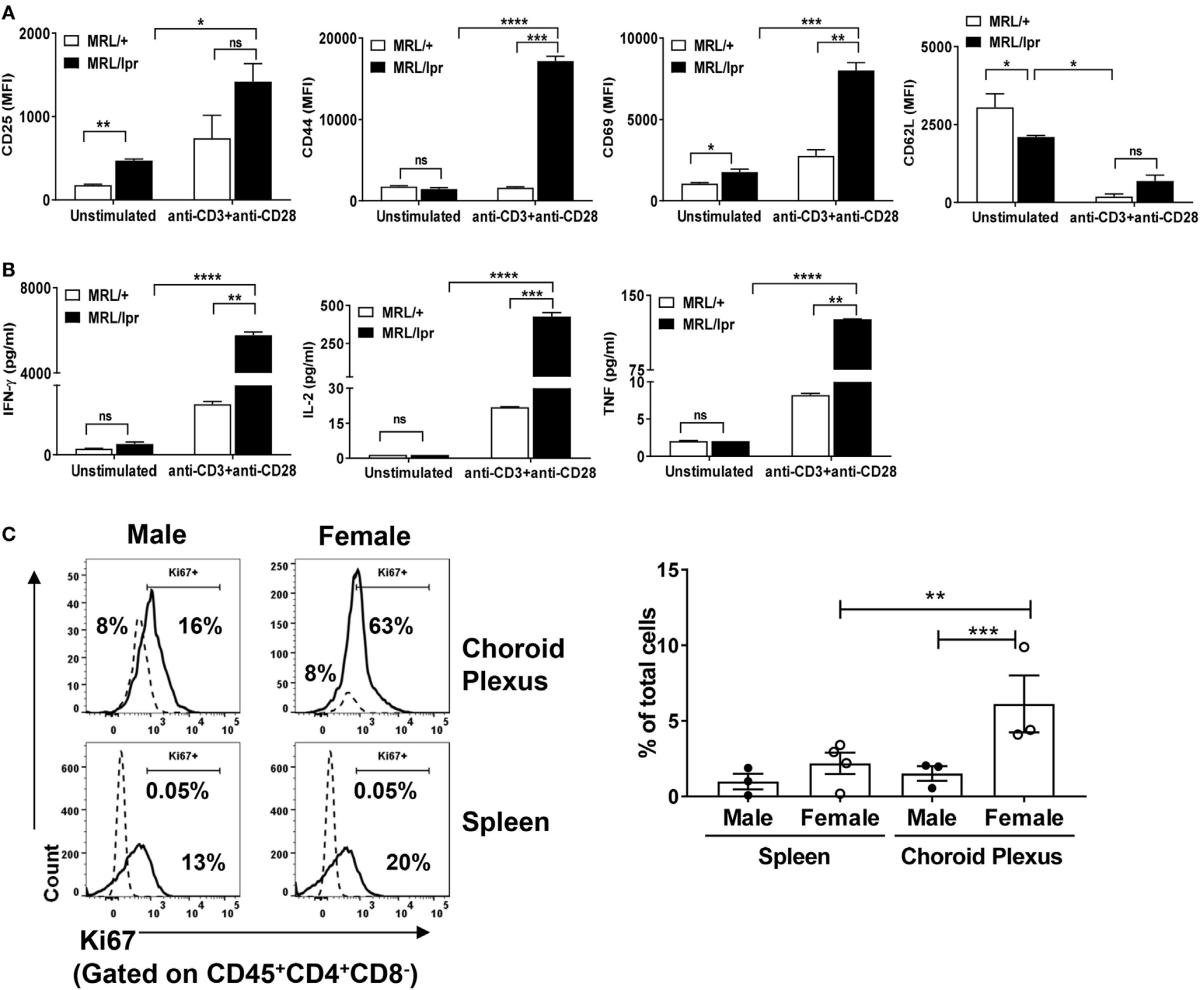
Brain infiltrating T cells are activated. Sorted CD4^+^ T cells from choroid plexuses of 16–18-week-old female MRL/+ (*n* = 3) and MRL/lpr (*n* = 5) were stimulated *in vitro* with anti-CD3 and anti-CD28. **(A)** Cells were analyzed for the expression of T cell activation markers: CD25, CD44, CD69, and CD62L. **(B)** Culture supernatants were analyzed for the quantification of different cytokines by LEGENDplex. Bars represent mean ± SEM of duplicate wells, and the statistical significance was determined by the Holm-Sidak method with alpha = 0.05. **(C)** CD45^+^CD4^+^CD8^−^ T cells from choroid plexus and spleen were stained for the expression of Ki67 (solid lines). Isotype control (dotted lines) were used to set positive and negative gates. Values in the histogram indicate percentage of parent (CD4) cells (left panel). The right panel shows the cumulative data depicting the percentage of Ki67^+^ in the total number of cells from spleen and choroid plexus of male (*n* = 3) and female (*n* = 3–4) mice. Data are represented as mean ± SEM of percentage of total cells. Each dot represents one mouse. *p*-Values were determined by one-way ANOVA. The *p*-values were calculated with an unpaired *t*-test. *p < 0.05, **p < 0.01, ***p < 0.001.

Next, we determined whether brain infiltrating CD4^+^ T cells in MRL/lpr mice have an activated phenotype. To address this, we sorted CD4^+^ T cells from CP tissue, and stained for the expression of T cell activation markers. We found that unstimulated brain derived CD4^+^ T cells from MRL/lpr mice had an enhanced activated phenotype as compared to MRL/+ controls, as evidenced by increased expression of CD25 and CD69 and decreased expression of CD62L (Figure 2A). Moreover, upon stimulation with anti-CD3 and anti-CD28, the expression of T cell activation markers in MRL/lpr mice was further significantly enhanced (Figure 2A). Thus, infiltrating CD4^+^ T cells from the CP of MRL/lpr mice have an inherently activated phenotype, which can be further enhanced by activating signals.

To further understand the functional potential of these T cells, we isolated CD4^+^ T cells from MRL/lpr CP, cultured them *in vitro* in the presence of anti-CD3 and anti-CD28 antibodies, and measured cytokine production. There was a significant increase in the production of IFN-γ and IL-2 in activated CD4^+^ isolated from MRL/lpr compared to those obtained from MRL/+ mice (Figure 2B). IL-4, IL-17, and IL-9 could not be detected. In addition, increased expression of TNF was also detected in MRL/lpr T cells, but at lower absolute concentrations (Figure 2B). These findings further support that brain infiltrating CD4^+^ T cells in MRL/lpr mice have a functionally activated phenotype.

Hyperactive lupus T cells have impaired tolerance due to altered intracellular signaling, leading to heightened proliferative potential (29). Therefore, we investigated whether the abnormal accumulation of brain infiltrating CD4^+^ T cells is due to enhanced proliferative capability. For examination of the proliferative potential, we stained CP CD4^+^ T cells for the expression of Ki67. To better display the findings in female MRL/lpr mice and further demonstrate the close association between the T cell phenotype and neuropsychiatric disease, the control strain used in the following experiments were male MRL/lpr mice, which display an attenuated neuropsychiatric phenotype (18). We found that besides being more extensively infiltrated by T cells, female MRL/lpr CP T cells showed significantly more expression of Ki67 as compared to male MRL/lpr mice (Figure 2C). This is consistent with our previous report that while male MRL/lpr mice do display a neuropsychiatric phenotype, it is delayed and less severe relative to female mice (18). It is important to emphasize that the expression of Ki67 was measured here without additional stimulation *in vitro*, supporting the notion that CP T cells in lupus receive substantial activation signals *in vivo*. To further correlate this observation with differences in the severity of systemic disease between female and male mice, we also compared Ki67 staining in spleen CD4^+^ T cells between the sexes and found similar results (Figure 2C). From these observations, we conclude that brain infiltrating CD4^+^ T cells in MRL/lpr mice not only have a functional activated phenotype, but also substantially increased proliferative capacity.

### Lupus CP Infiltrating Lymphocytes Include IL-21 and IFN-γ Secreting T Cells

T cell signaling abnormalities account for the B cell hyperactivity that drives lupus manifestations (5). Th subsets such as Th1, Th17, and T_FH_ cells, secreting IFN-γ, IL-17, and IL-21, respectively, play an important role in the progression of SLE (30). To identify the specific Th subsets associated with neuropsychiatric lupus manifestations, we performed intracellular cytokine staining for IFN-γ (Th1), IL-17 (Th17), and IL-21(T_FH_) on CD4^+^ T cells isolated from the CP of MRL/lpr and MRL/+ mice.

**Figure 3:**
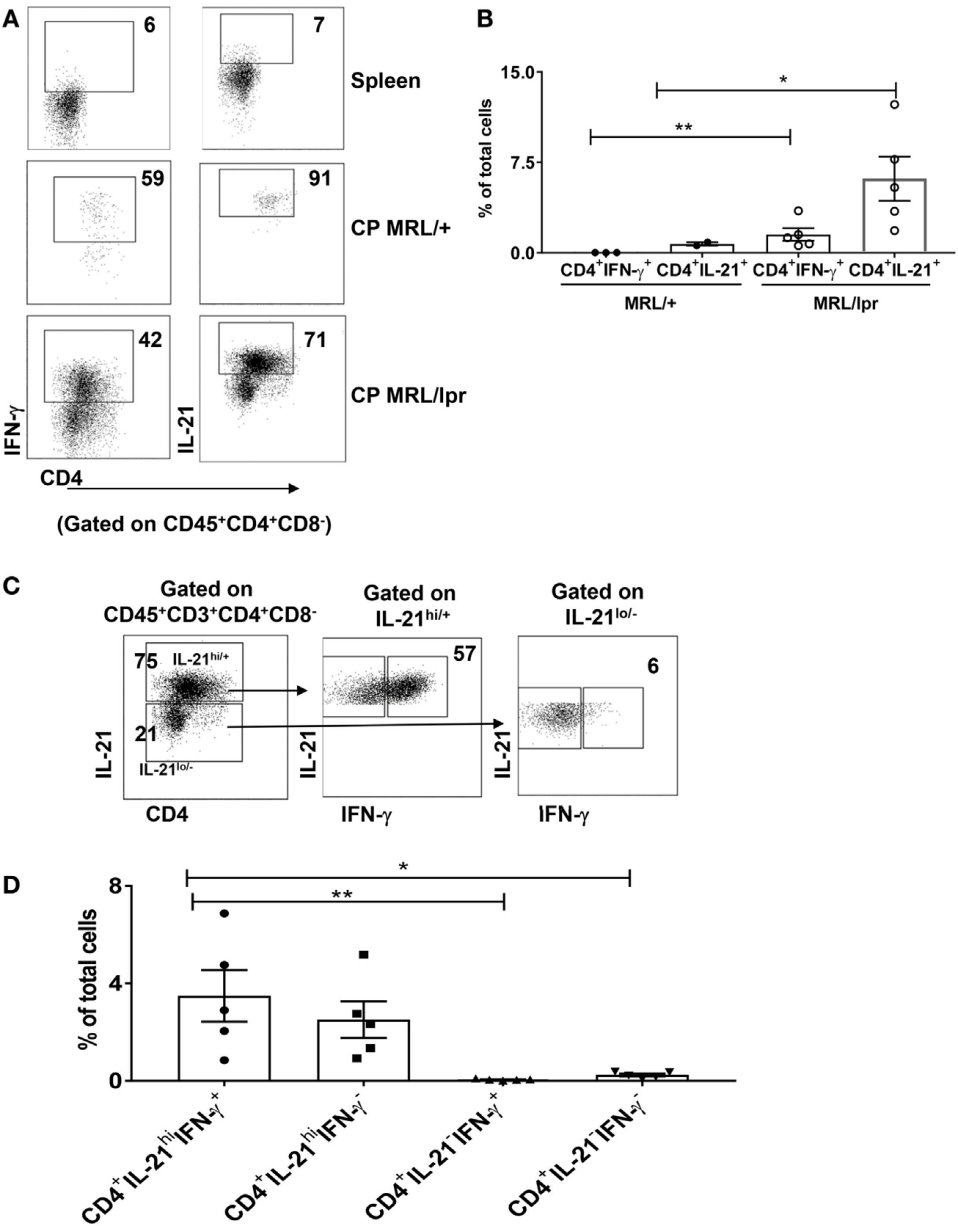
T cells infiltrating the brain predominantly secrete IFN-γ and IL-21. **(A)** Single cell suspensions from choroid plexus (CP) of 16–18-week-old female MRL/+ (*n* = 2–3) and MRL/lpr (*n* = 5) mice were stimulated with PMA and ionomycin for 6 h, and stained for intracellular IFN-γ and IL-21. Values in the FACS plots represent percentage of parent (CD4^+^) population. **(B)** Bars represent mean ± SEM of the percentage of CD4^+^ IFN-γ^+^ cells and CD4^+^ IL-21^+^ cells from the total number of cells in the CP. **(C)** FACS plots showing relative expression of IFN-γ in IL-21^hi^ and IL-21^lo/neg^ cells. Values indicate percentage of parent population (indicated above each plot). **(D)** Graphs depict mean ± SEM of the percentage of CD4^+^IFN-γ^+^IL-21^+^, CD4^+^IFN-γ^−^IL-21^+^, CD4^+^IFN-γ^+^IL-21^−^, and CD4^+^IFN-γ^−^IL-21^−^ cells in the total number of cells in the CP of MRL/lpr mice. Each dot represents one mouse. PD1 and CXCR5 negative spleen T cells were used to set the negative gates for IFN-γ and IL-21 in this figure. The *p*-values were determined by unpaired two-tailed *t*-test with Welch’s correction. *p < 0.05, **p < 0.01.

We found that the CD4^+^ T cells that infiltrate the brains of MRL/lpr mice contained significant populations of CD4^+^IFN-γ^+^ as well as CD4^+^IL-21^+^ cells (Figures 3A,B). In contrast, we could not identify any significant absolute numbers of IL-4 or IL-17 secreting CD4^+^ T cells in these mice (Figure S2 in Supplementary Material). Furthermore, as compared to MRL/+, MRL/lpr CP did not overexpress IL-4 or IL-17 by RNA-sequencing (data not shown). Therefore, we concluded that neuropsychiatric disease manifestations in MRL/lpr lupus mice are primarily associated with CP localization of IFN-γ and IL-21 positive CD4^+^ T cells. We further analyzed brain infiltrating CD4^+^ T cells by costaining for both IFN-γ and IL-21, and found that most cells were IL-21^hi/+^, although IL-21^lo/−^ cells were present as well (Figure 3C). Interestingly, there were two distinct IL-21^hi/+^ sub-populations of almost similar size, IL-21^hi/+^ IFN-γ^+^ and IL-2^hi/+^ IFN-γ^−^ (Figure 3C middle panel, 3D). These two subpopulations were also observed in IL-21^lo/−^ cells (Figure 3C right panel, 3D), but the absolute representation of both IL-21^lo/−^ IFN-γ^+^ and IL-21^lo/−^ IFN-γ^−^ T cells was small (Figure 3D). These findings lead us to conclude that neuropsychiatric manifestations are associated with a subset of CD4^+^ T cells that principally secretes IL-21 and IFN-γ.

### CP Infiltrating CD4^+^ T Cells Have a T_FH_-Like Phenotype

**Figure 4:**
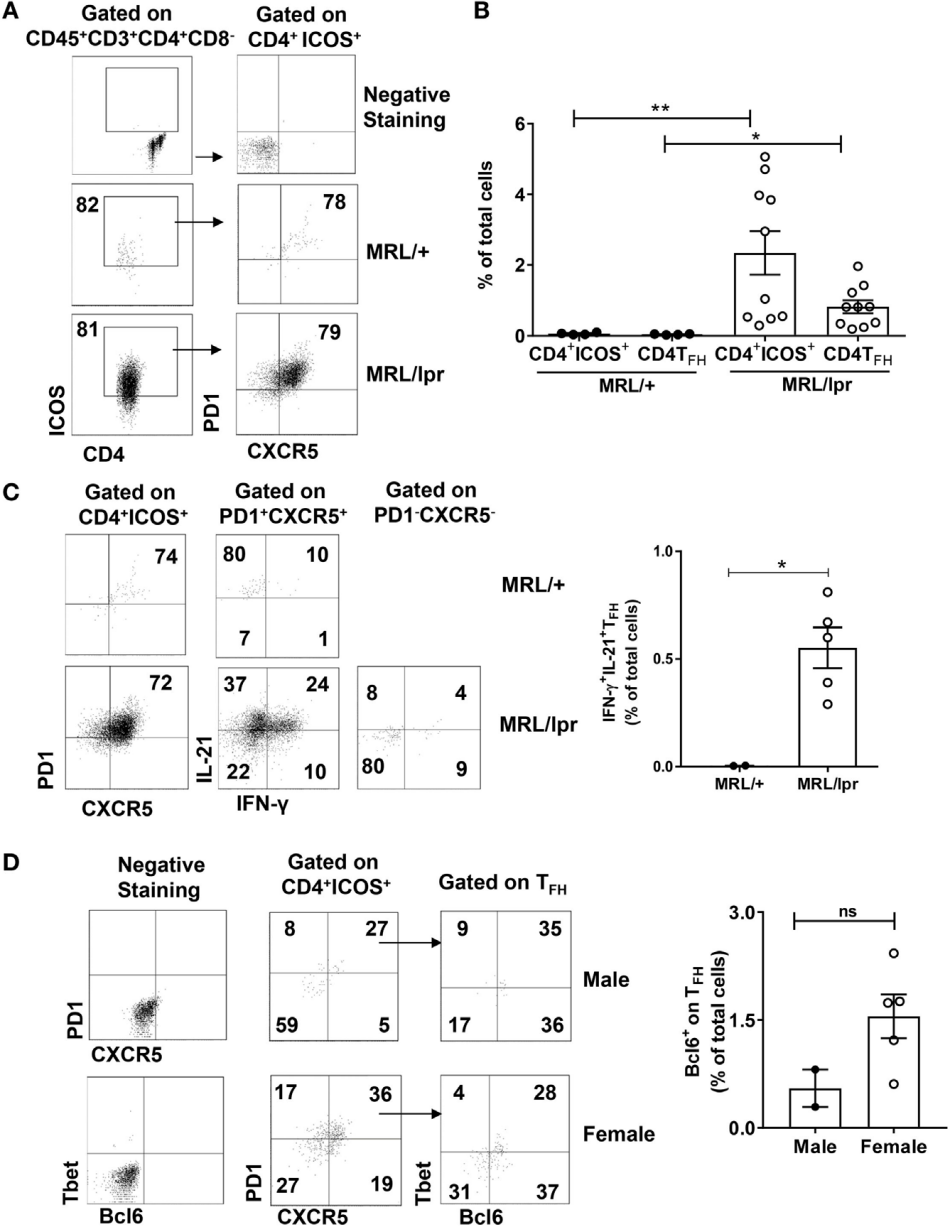
Infiltrating T cells have a T_FH_ phenotype. Single cell suspensions from choroid plexus of MRL/+ (*n* = 4), MRL/lpr male (*n* = 2), and MRL/lpr female (*n* = 10) mice were stained for the expression of CD4, ICOS, PD1, and CXCR5. **(A)** Comparative FACS plots showing CD4^+^ICOS^+^PD1^+^ CXCR5^+^ T_FH_ cells. Values in the plots represent percentage of parent population (indicated above each plot). Negative staining controls are shown in the top two panels. **(B)** Quantitative data representing mean ± SEM of the total percentages of CD4^+^ICOS^+^ and CD4^+^ICOS^+^PD1^+^ CXCR5^+^ T_FH_ cells. Each dot represents one mouse. **(C)** Representative FACS plots showing the expression of IL-21 and IFN-γ in PD1^+^CXCR5^+^ T_FH_ cells and PD1^−^CXCR5^−^ T cells. Values in the plots represent percentage of parent population (indicated above each plot). Bar graphs indicate mean ± SEM of the total percentages of IL-21^+^ IFN-γ^+^ T_FH_ cells (right panel). **(D)** Comparative FACS plots depicting the expression of Bcl6 on T_FH_ cells (left panel). Values in the plots represent percentage of parent population (indicated above each plot). Bar graphs indicate mean ± SEM of the total percentages of Bcl6^+^ T_FH_ cells (right panel). Negative staining controls are shown in the left two panels. The *p*-values were determined by unpaired two-tailed *t*-test with Welch’s correction. *p < 0.05, **p < 0.01.

Both IL-21 and IFN-γ have been implicated in lupus pathogenesis (31, 32). IL-21 is primarily secreted by T_FH_ cells that are phenotypically defined as CD4^+^ICOS^+^PD1^+^CXCR5^+^ (33). It has also been shown in inflammatory bowel disease that T_FH_ cells coproduce IFN-γ in addition to IL-21 (34). Moreover, excessive production of IFN-γ promotes accumulation of T_FH_ cells and the formation of germinal centers (35). Therefore, we investigated whether the CD4^+^ T cells infiltrating the brain of MRL/lpr mice have a T_FH_ phenotype. We found that a major subset of CP CD4^+^ T cells had upregulated ICOS (Figures 4A,B) and that CD4^+^ICOS^+^ cells also had significant expression of PD1 and CXCR5, thereby qualifying them as T_FH_ cells (Figures 4A,B).

To further confirm that neurological manifestations in MRL/lpr mice are accompanied by the accumulation of IFN-γ and IL-21 producing CD4^+^ T_FH_ cells, we analyzed the expression of these cytokines along with the signature T_FH_ markers. We found that CD4^+^ICOS^+^PD1^+^CXCR5^+^ cells had significant expression of IFN-γ and IL-21 (Figure 4C; Figure S3 in Supplementary Material). To further establish that these cells are bona-fide CD4^+^ T_FH_ cells and not Th1 cells, we studied the expression of the transcription factors Bcl6 and Tbet. We found that CD4^+^ICOS^+^PD1^+^CXCR5^+^ cells expressed Bcl6 as well as Tbet (Figure 4D). Although the difference between male and female mice was not significant, this experiment does clearly demonstrate a cell population expressing Bcl6 without Tbet coexpression infiltrating the CP of MRL/lpr mice. The prominent T_FH_ phenotype present in the CP of MRL/lpr mice was supported by RNA-sequencing studies, which demonstrated highly significant differential expression of IL-21, ICOS, PD1, and CXCR5 (data not shown). We also studied GATA3 and RORγt, other key transcription factors in T cell subsets, but these were negative, both by intracellular staining and by RNA-sequencing (data not shown). These observations lead to a conclusion that a major subset of CP infiltrating T cells are IFN-γ and IL-21 secreting CD4^+^ T_FH_ cells.

### A Diminished Regulatory Mechanism Is Associated with NPSLE

**Figure 5:**
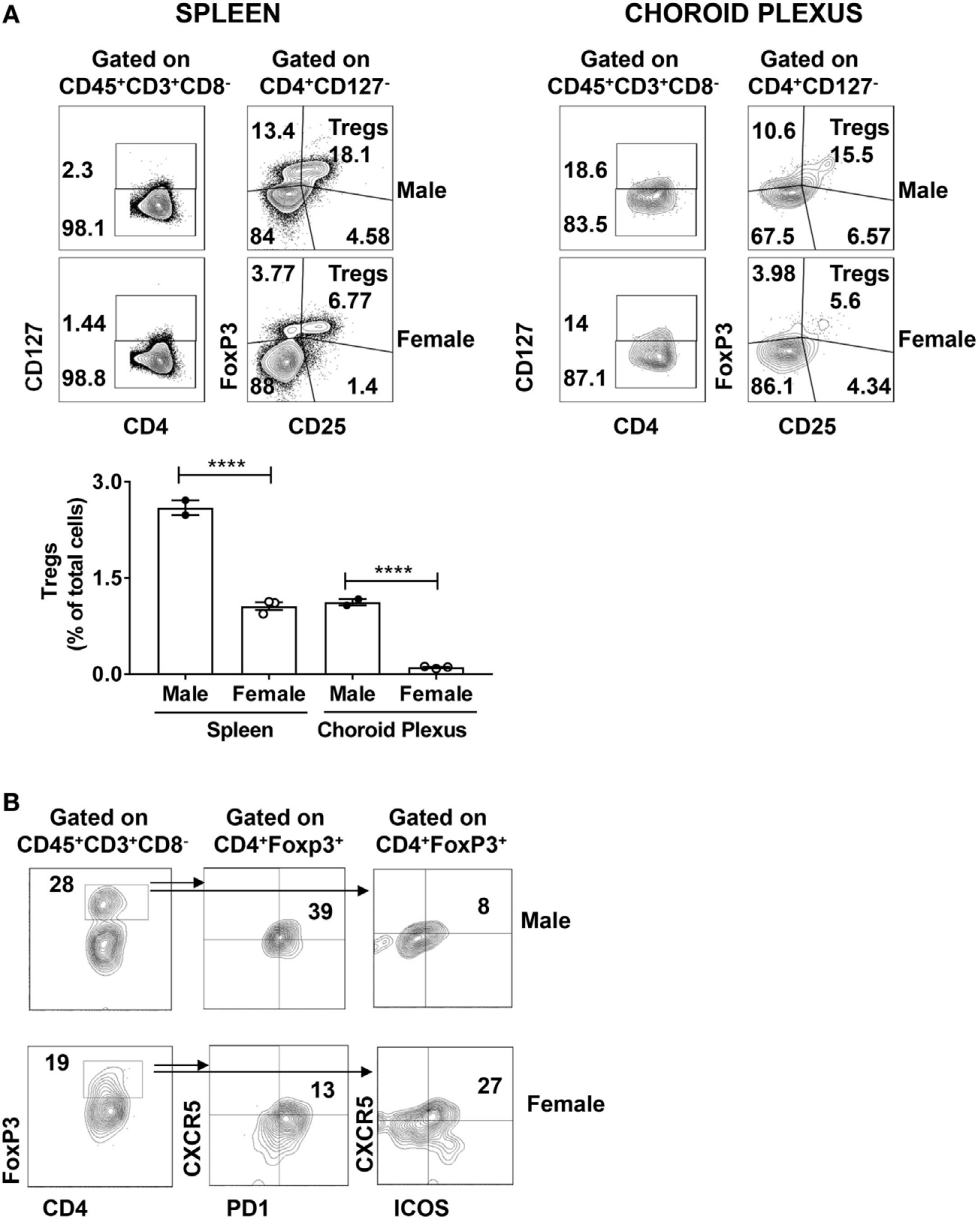
Regulatory T cell populations are decreased in the brains of MRL/lpr mice. **(A)** Spleen and choroid plexus of MRL/+ and MRL/lpr mice were stained for the expression of CD4^+^CD127^−^CD25^+^ FoxP3^+^ T regulatory cells (Tregs). Shown are the representative FACS plots of male (*n* = 2) and female (*n* = 3) MRL/lpr spleen (left) and choroid plexus (right). Values in the plots represent percentage of parent population (indicated above each plot). Bar graph represents mean ± SEM of the total percentages of Tregs in male and female spleen and choroid plexus, respectively. Each dot represents one mouse. The *p*-values were determined by one way ANOVA with Sidak’s multiple comparison test. ****p < 0.0001. **(B)** Choroid plexus cells from MRL/lpr male (*n* = 2) and MRL/lpr female (*n* = 5) mice were stained and analyzed for the presence of T follicular regulatory cells. FACS plots depict the expression of CXCR5^+^PD1^+^ (middle panel) or CXCR5^+^ICOS^+^ (right panel) cells on CD4^+^FoxP3^+^ (left panel) cells. Values in the plots represent percentage of parent population (indicated above each plot).

Systemic lupus erythematosus has been linked to abnormally downregulated regulatory control that can be attributed to reduced percentages of regulatory T cells (Tregs) (36). Tregs are classically defined as CD4^+^CD127^−^CD25^+^FoxP3^+^ cells that can secrete IL-10 and TGF-β. Moreover, Tregs reciprocally regulate pathogenic IFN-γ+ populations (37, 38). To establish whether reduced numbers of CD4^+^ Tregs might be contributing to the pathogenesis of murine NPSLE, we stained CP derived T cells from age-matched male and female MRL/lpr mice for the expression of CD4^+^CD127^−^CD25^+^FoxP3^+^. We found a significant reduction in CD4^+^CD127^−^FoxP3^+^ cells in the CP of female mice (Figure 5A, upper right panel). For comparison, we also stained spleens of the same male and female MRL/lpr mice for the presence of Tregs. Spleens from sick female MRL/lpr mice also had decreased Treg numbers (Figure 5A, upper left panel), in line with previous studies indicating that the T regulatory axis is defective in lupus (39, 40).

Using an alternative gating strategy, we found significant expression of FoxP3 on CD4^+^ T cells alone (Figure 5B left panel). This led us to speculate that there may be another FoxP3 mediated regulatory population that is involved. It is well known that the CD4^+^ T_FH_ mediated GC reaction is controlled by a suppressive population of cells known as Tfr (41, 42). Tfr cells are defined as CD4^+^ PD1^+^CXCR5^+^ FoxP3^+^ cells that can coexpress Bcl6. In addition, IL-21 aids in B cell stimulation and GC formation by mediating the suppression of Tfr (43). Moreover, altered T_FH_:Tfr ratios are implicated in several autoimmune diseases (44, 45). We did identify Tfr cells among brain infiltrating T cells, as demonstrated by ICOS, PD1, and CXCR5 positivity in CD4^+^FoxP3^+^ cells (Figure 5B; middle and right panel). Moreover, there were some differences between male and female mice in marker distribution (Figure 5B). However, since the absolute number of Tfr cells in each sex was very small, no firm conclusion can be reached at this time regarding a possible contribution of abnormalities in Tfr numbers to the CP T cell or neuropsychiatric disease phenotype.

## Discussion

Aberrant T cell activity plays an integral role in lupus pathogenesis. An altered cytokine milieu in lupus not only results in altered T cell activation but also influences subset differentiation. The subsequent imbalance between the pathogenic and the regulatory T cell subsets leads to abnormal immunity and organ injury. In particular, DN T cells, Th17 cells, T_FH_ cells, and Tregs have all been implicated in lupus pathogenesis (46, 47). However, detailed characterization of brain infiltrating T cells in NPSLE, a central manifestation of SLE with major prognostic significance, has not been performed to date.

Compromised integrity of brain barriers in lupus allows for the passage and homing of circulating lymphocytes and autoantibodies to the brain, which contribute to neuropsychiatric symptoms (48, 49). In the present study, we identify and characterize T cells infiltrating the brain in MRL/lpr mice. Our findings indicate that the majority of infiltrating T cells are not actually DN T cells, but rather single positive CD4^+^ or CD8^+^ T cells. Moreover, the infiltrating T cells are functionally activated and have enhanced expression of CD44, thereby contributing to the effector phenotype. This finding is consistent with previous reports that T cells from peripheral blood mononuclear cells and renal biopsies of SLE patients have a pronounced CD44 mediated signaling cascade that may allow these cells to migrate abnormally (50). A significant population of memory T cells were also found in the brain infiltrating lymphocytes. This implies that there is enhanced *in vivo* stimulation of T cells in NPSLE, likely by self-antigens, that results in the accumulation of memory T cells. The enhanced proliferation of CP as compared to splenic T cells is also consistent with specific brain accumulation and local activation of these cells, rather than simply reflecting the systemic T cell pool. However, whether these memory T cells have decreased proliferative ability and increased apoptotic ability comparable to that reported in circulating SLE memory T cells is as yet unclear (51). Furthermore, it also remains to be determined whether such antigenic stimulation occurs locally (i.e., in the brain) or systemically. Although the antigenic specificity of the CP infiltrating T cells is not known at this time and may not be easy to identify, this is an interesting and important question that will need to be addressed in future studies.

The control mice used for our studies merit discussion. The congenic MRL/+ mice only differ from the lupus prone MRL/lpr strain in a CD95 mutation, yet at 4–5 months of age do not display CP infiltration, neuropsychiatric deficits, or indeed systemic autoimmunity. The MRL/+ therefore is a commonly used control strain in lupus studies. Nevertheless, to better illustrate some of our findings in female mice and further demonstrate the close association between the T cell phenotype and neuropsychiatric disease, we preferred in some experiments to use a strain from which sufficient CP T cells could still be obtained for study. We found that male MRL/lpr mice demonstrated an attenuated T cell phenotype as compared to females, a finding consistent with the female bias present for both systemic and neuropsychiatric disease in this strain (18, 19).

Possible fates of differentiation of naive T cells in lupus include development into T_FH_ cells under the transcriptional regulation of Bcl6 to interact with B cells to produce autoantibodies, or maturation into effector Th17 subsets (28). Interestingly, the brain infiltrating T cells were primarily comprised of T_FH_, whereas evidence for Th17 cells was not found either at the level of cytokines or transcriptional factors. The expression of signature surface markers (ICOS, CXCR5, and PD1), transcription factor (Bcl6), and IL-21 clearly defined a T_FH_ phenotype. To the best of our knowledge, this is the first report identifying T_FH_ in brain infiltrating T cells in NPSLE, although a pathogenic role of T_FH_ cells in lupus progression is already suggested (52). Remarkably, many T_FH_ cells were found to coexpress both IL-21 and IFN-γ. It is known that secondary TCR stimulation of activated T cells induces IL-21 production (53); the secretion of large amounts of IL-21 by CP infiltrating CD4 T cells may be due to overt local activation of T_FH_ cells in the CP. In addition, it has been shown that a distinct subset of T_FH_ cells coexpressing IFN-γ and IL-21 drives inflammatory bowel disease (34), and has been recently described in lupus albeit not in the brain (54). Along the same lines, we believe that a unique population of T_FH_ cells, coexpressing both IL-21 and IFN-γ, is driving the neuropsychiatric manifestations of NPSLE. The presence of T_FH_ cells also suggest that the lymphocytic infiltrates present in the brain of MRL/lpr lupus mice may be organized in the form of follicular structures, a possibility supported by a recent pilot study (55). However, additional studies will be required to demonstrate whether these T_FH_ cells are actually pathogenic and whether they have the ability to help brain infiltrating B cells produce autoantibodies to nuclear and/or brain antigens.

Patients with active SLE have fewer CD4^+^ Tregs as compared with healthy controls due to the reduced levels of FoxP3 expression (37). Also, it has been shown that SLE effector T cells are resistant to the effect of Tregs and that increased production of IL-6 leads to Treg inhibition (56, 57). Hence, a regulatory mechanism is disrupted in lupus leading to hyperactivation of pathogenic T cell subsets. Akin to the systemic disease, we found that the brain infiltrating T cells did not contain any significant signature CD4^+^ T regulatory population (CD4^+^CD127^−^CD25^+^FoxP3^+^). However, this was not due to reduced expression of FoxP3, since substantial expression of FoxP3 was found on CD4^+^ T cells. As there was a considerable population of T_FH_ in brain infiltrates, we considered whether FoxP3 expressing CD4^+^ T cells may conform to a Tfr phenotype that regulates T_FH_ mediated B cell activation (58). Following an established gating strategy to identify Tfr (59), we did find expression of ICOS, PD1, and CXCR5 on CD4^+^FoxP3^+^ cells. However, due to low cell numbers definitive inferences concerning the contribution of Tfr cells are premature, and their role in NPSLE remains to be determined.

Brain biopsy is only rarely done as part of the workup of patients with suspected neuropsychiatric lupus. Moreover, in our experience, autopsy tissue is difficult to obtain and quite rare even in centralized tissue banks, especially from lupus patients without major confounding factors (secondary causes of brain disease, prolonged immunosuppressive treatment). Nevertheless, despite the challenges in collecting sufficient tissues for study, it will be important to make a serious effort toward human validation of these observations.

In conclusion, the present study identifies and characterizes T cells that infiltrate exclusively in the brain CP in a well-established murine NPSLE model. We describe a prominent CD4^+^ T_FH_ like subset that coexpresses IFN-γ and IL-21 in the MRL/lpr strain, which is associated with and may play a role in the progression of neuropsychiatric manifestations. Although our findings are surely consistent with a pathogenic role for CP infiltrating T cells (reflected in the differences between MRL/lpr and MRL/^+^ mice, and the temporal development of cognitive deficits in MRL/pr mice only once brain lymphocytic infiltration is detected), these do not yet constitute direct evidence. Therefore, the pathogenic nature of these brain infiltrating T cells and their ability to provide B cell help needs to be conclusively established. It is also important to investigate whether these infiltrating CD4 T_FH_ can precipitate pathogenic manifestations in predisease (young) MRL/lpr mice or congenic MRL/^+^ controls (adoptive transfer). Alternatively, this question can also be addressed using genetic approaches. While whether a similar T cells population or subset can be identified in human NPSLE brain tissue remains to be seen, characterizing the TCR repertoire and determining the antigen specificity of these brain infiltrating T cells can further illuminate the contribution of T cells to the pathogenesis of NPSLE, and may also facilitate the design of effective new therapeutic strategies to treat this challenging manifestation of lupus.

## Ethics Statement

All animal studies were performed under protocols approved by the Institutional Animal Care and Use Committee of the Albert Einstein College of Medicine.

## Author Contributions

SJ, AS, FM, and CP conceived and designed the experiments. SJ performed the reported studies. SJ, FM, and CP analyzed the data. SJ, AS, FM, and CP wrote and/or edited the article and approved the final submitted version.

## Conflict of Interest Statement

The authors declare that the research was conducted in the absence of any commercial or financial relationships that could be construed as a potential conflict of interest.

## Abbreviations

SLE, systemic lupus erythematosus; NPSLE, neuropsychiatric systemic lupus erythematosus; CNS, central nervous system; DN, double negative; CP, choroid plexus; FcR, Fc receptor; Th, T helper; T_FH_, T follicular helper cells; Tregs, T regulatory cells; Tfr, T follicular regulatory cells.
