# Supplementary figures and images for: A Distinct T Follicular Helper Cell Subset Infiltrates the Brain in Murine Neuropsychiatric Lupus
Source: Front Immunol. 2018 Mar 13;9:487. doi: 10.3389/fimmu.2018.00487 (PMC5859360; doi:10.3389/fimmu.2018.00487)

**Figure S1**

**A**

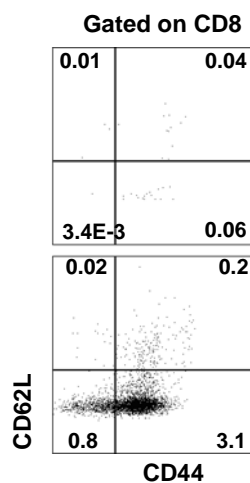

**B**

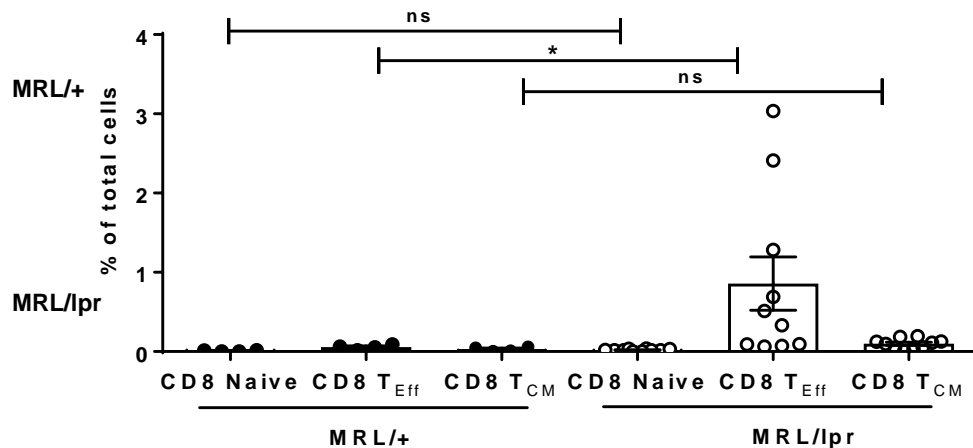

**Figure S2**

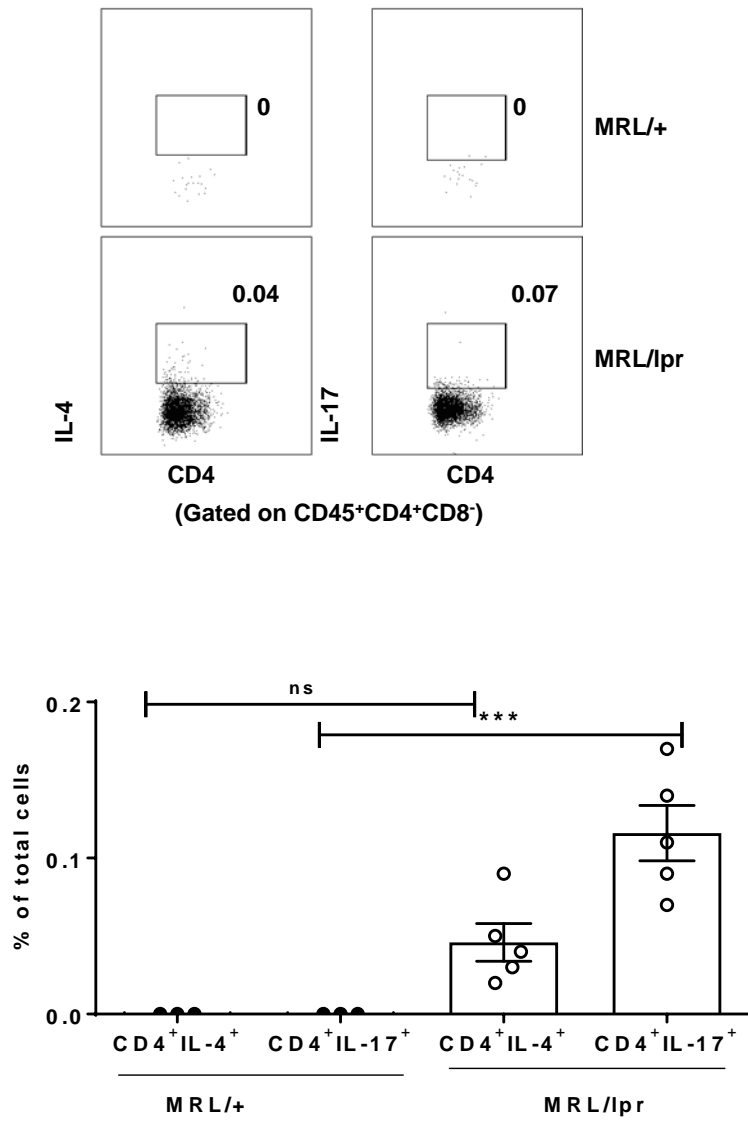

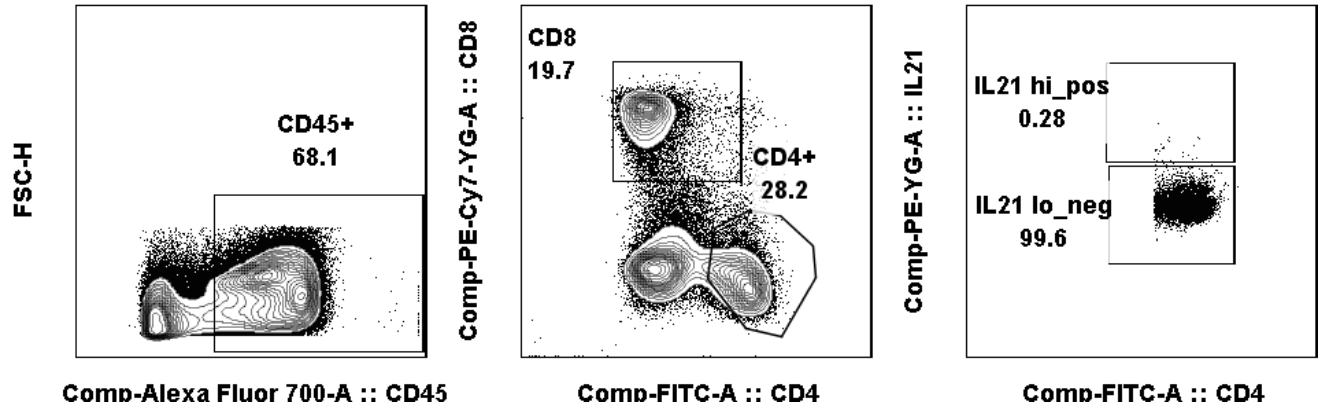

Supplement: Supplementary file 1 [file image_1.PDF]
